# Delayed electron emission in strong-field driven tunnelling from a metallic nanotip in the multi-electron regime

**DOI:** 10.1038/srep35877

**Published:** 2016-10-27

**Authors:** Hirofumi Yanagisawa, Sascha Schnepp, Christian Hafner, Matthias Hengsberger, Dong Eon Kim, Matthias F. Kling, Alexandra Landsman, Lukas Gallmann, Jürg Osterwalder

**Affiliations:** 1 Institute for Quantum Electronics, ETH Zürich, CH-8093 Zürich, Switzerland; 2 Physik-Institut, Universität Zürich, CH-8057 Zürich, Switzerland; 3 Max Planck Institute of Quantum Optics, D-85748 Garching, Germany; 4 Max Planck POSTECH/KOREA Res. Init., Pohang, 37673, South Korea; 5 Physics Department, Ludwig-Maximilians-Universität München, D-85748 Garching, Germany; 6 Laboratory for Electromagnetic Fields and Microwave Electronics, CH-8092 Zürich, Switzerland; 7 Department of Physics, POSTECH, Pohang, 37673, South Korea; 8 Institute of Applied Physics, University of Bern, CH-3012 Bern, Switzerland

## Abstract

Illuminating a nano-sized metallic tip with ultrashort laser pulses leads to the emission of electrons due to multiphoton excitations. As optical fields become stronger, tunnelling emission directly from the Fermi level becomes prevalent. This can generate coherent electron waves in vacuum leading to a variety of attosecond phenomena. Working at high emission currents where multi-electron effects are significant, we were able to characterize the transition from one regime to the other. Specifically, we found that the onset of laser-driven tunnelling emission is heralded by the appearance of a peculiar delayed emission channel. In this channel, the electrons emitted via laser-driven tunnelling emission are driven back into the metal, and some of the electrons reappear in the vacuum with some delay time after undergoing inelastic scattering and cascading processes inside the metal. Our understanding of these processes gives insights on attosecond tunnelling emission from solids and should prove useful in designing new types of pulsed electron sources.

A number of studies have clarified the intriguing characteristics of the electron emission processes that occur when a nano-sized metallic tip is illuminated with ultrashort laser pulses. Plasmonic effects enhance optical electric fields at the tip apex^1^, leading to spatial confinement and enabling control of the electron emission on a nanometer scale^2^,^3^,^4^. The strength of the laser field at the emission site determines the emission mechanism, and the temporal confinement of the emitted electron pulses depends on the mechanism. For relatively weak laser fields, in combination with a strong DC voltage applied to the tip, electrons excited by multi-photon absorption tunnel through the surface barrier (DC tunnelling) or are emitted over the barrier as illustrated in Model A in Fig. 1(a)^2^,^3^,^4^,^5^,^6^,^7^,^8^,^9^,^10^,^11^. These processes are insensitive to the laser phase and generate femtosecond electron pulses^6^,^7^,^8^,^9^. On the other hand, very strong laser fields largely modify the surface barrier and drive direct tunnelling emission through the barrier as shown in Model B (AC tunnelling), producing attosecond coherent electron waves^9^,^10^,^12^,^13^,^14^,^15^,^16^,^17^,^18^,^19^,^20^,^21^. This laser-driven AC tunnelling emission - also termed optical field emission - has become the subject of intense research in ultrafast science.

In the strong field regime, the generated coherent electron waves show an intriguing attosecond phenomenon, so-called re-scattering emission^14^,^15^,^16^,^22^. In the re-scattering emission process, the electrons produced by laser-driven tunnelling emission are driven back to the surface by the oscillating laser fields where some of the electrons are elastically backscattered at the very surface of the tip. They contribute to the prompt emission as indicated by orange arrows in Fig. 1(b). The re-scattering process generates high energy electrons from elastic re-scattering off the tip surface^16^. According to theoretical work, up to 20 percent of the emitted electrons will be elastically backscattered at the surface while the majority of electrons are scattered in the forward direction, i.e. into the tip^22^. Now the question arises: where do the majority of the electrons go? Knowing the dynamics of these electrons is crucial for the design of a practical electron source based on laser-driven tunnelling emission. These electrons are considered to travel inside the metal and some of them should still come back to the surface with some temporal delay after multiple elastic and inelastic scattering as indicated by green arrows in Fig. 1(b)^22^. In the present work, we have focused into this particular channel, and we found that such a delayed emission can be distinguished by its characteristic energy distribution when multi-electron effects, namely space charge effects due to Coulomb interaction between the electrons during electron propagation through the vacuum, are strong. The multi-electron effects are impotant even for a few electrons per pulse in some cases^23^. This requires a critical difference in the experimental conditions compared to previous studies where the multi-electron effects were suppressed by either limiting the number of electrons below one electron per pulse^14^,^15^,^16^ or by using very sharp tips with radii of curvature of approximately 10 nm and moderate DC voltage bias^17^,^18^,^19^,^20^,^21^; in the latter case, multi-electron effects become less significant because of rapidly diverging trajectories and stronger field enhancement^24^.

Here we used much blunter tungsten tips with radii of curvature of approximately 100 nm and higher DC fields on the tip apex, realizing a case where the multi-electron effects are significant. In fact the conditions are in a similar regime as in a recent study of near-field enhanced electron emission from dielectric nanospheres excited with few-cycle laser fields, where the multi-electron effects were found to strongly affect the electron energy distribution^25^,^26^. In our experiment, using a setup as schematically drawn in Fig. 1(b), the emission spectra produced by few-cycle laser pulses from the tip apex show a plateau region spreading over several tens of eV (Fig. 1(c)) and a pronounced peak at the low-energy end when the laser intensity is increased to high values where several hundred electrons are emitted by each pulse. Analysing these data based on the three model scenarios of Fig. 1(a) with including the multi-electron effects, we find that this low-energy peak can only be produced by delayed electron emission, because all electrons emitted promptly within the duration of the light pulse contribute to the plateau or its high-energy tail. We present a simple delayed emission model that reproduces our observations very well. In our model, we assume the electrons driven back into the metal travel a distance of the order of twice the inelastic mean free path inside the metal before reappearing in the vacuum. At electron energies of about 5 eV above the Fermi energy *E*_*F*_, inelastic mean free path lengths are of the order of 10 nm^27^ (lower energy values for inelastic mean free paths were received via private communication), which can lead to delays of the order of a few to even tens of femtoseconds with respect to the prompt emission. The number of electrons that are subject to this delayed emission depends strongly on the initial impact energy on the surface and consequently on the initial emission mechanism. As we will show below, the delayed emission channel becomes strong only for the laser-driven tunnelling emission process, namely for Models B or C. Our simulations suggest that the low energy peak can serve as a smoking gun for the massive contribution of the tunnelling emission at higher laser intensities.

## Experimental results

The experimental energy spectra for 7 fs pulses at different laser intensities are shown in Fig. 2(a), where a smooth transition of the emission mechanism from the weak-field to the strong-field regime is observed. At the lowest laser intensity, a peak associated with two-photon photoemission (2PPE) dominates the spectrum, which is typical for the weak-field regime^6^,^7^. With increasing laser intensity, the peak grows in intensity and its maximum gradually moves towards slightly lower energies. Concurrently, a plateau feature with nearly constant intensity grows sideways towards higher energies. Gray arrows underline these two general trends. This behaviour is entirely different from that in the weak-field regime^6^,^7^. To analyze their evolution further, the peak and plateau intensities are plotted as a function of laser peak intensity in a log-log plot in the left panel of Fig. 2(d) (open symbols). The plateau first appears at a laser intensity of about 4 × 10^12^ W/cm^2^, and remains at almost constant emission level for higher intensities. At low laser intensities where the 2PPE spectrum dominates, the amplitude of the low-energy peak grows rather gentle but steeply after a pronounced upward kink indicated by a red arrow. In the right panel of the figure, another data set for a slightly lower DC voltage is shown. In this case, the tendency is similar, but the 2PPE peak shows two kinks (green and red arrows), with a steeper rise at the lowest laser powers. In order to illustrate the evolution of this feature towards lower laser peak intensities, we have added a similar data set measured with 70 fs laser pulses (filled triangles)^6^,^7^ to the left panel. Most of these data lie strictly on a straight line with a slope of 2 (dashed line), characteristic of 2PPE. The evolution, however, deviates from the slope of 2 around the green arrow where we observed the gentle slope with the 7 fs laser pulses; the deviation makes a feature similar to the first kink of the right panel. If the peak simply originated from the 2PPE excitations, the evolution would show a straight line with a slope of 2. Therefore, these kinks are strong indicators of changes in the emission process.

## Numerical analysis

**Figure 1:**
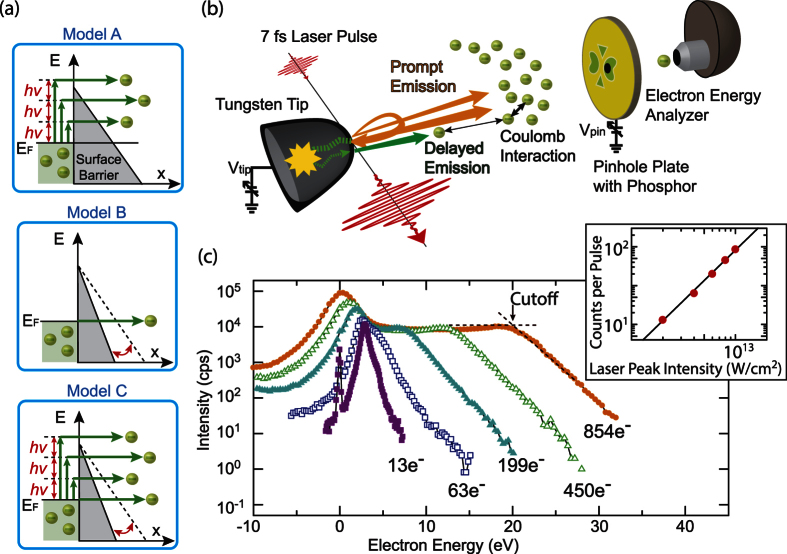
Conceptual diagrams of the laser-induced electron emission, electron spectra and experimental setup. (**a**) Illustration of different emission mechanisms, referred to as Models A to C, discussed in this work. (**b**) Schematic representation of the experimental setup. A tungsten tip oriented towards the [011] crystal direction is mounted in a vacuum chamber. 7 fs laser pulses (centre wavelength: 830 nm; repetition rate: 80 MHz) are focused onto the apex of the tip. The beam waist at the tip apex is estimated to be approximately 3.5 *μ*m in diameter. A pinhole plate covered with a phosphor is installed in front of the tip to observe the field emission pattern and to define the emission sites for spectroscopic measurements. A hemispherical electrostatic analyzer was used to measure the energy spectra. (See Methods Section for details). Different emission processes under strong fields are indicated by orange and green arrows. (**c**) Evolution of electron emission spectra with average laser intensities (values are 2 × 10^12^ W/cm^2^, 4 × 10^12^ W/cm^2^, 6 × 10^12^ W/cm^2^, 8 × 10^12^ W/cm^2^ and 10 × 10^12^ W/cm^2^, which correspond to laser powers of 18 mW, 36 mW, 54 mW, 72 mW and 90 mW, respectively), with electron numbers per pulse indicated for each curve. Intensities are given in a logarithmic scale. The electron numbers per pulse were plotted as a function of laser peak intensities in the inset, where the solid line is fitting curves with power functions; exponents are 2.6.

An intuitive interpretation of the spectra appears difficult because of the multi-electron effects; several hundred electrons per pulse were emitted from the tip apex at the higher laser intensities, where electron numbers per pulses increase with the laser intensities as shown in Fig. 1(c). Hence, to understand the physics of the observed data, we have simulated electron trajectories in the vacuum with taking the multi-electron effects into account. Initial conditions of electron emission were determined based on Monte Carlo sampling from the separately calculated emission current distribution on the tip apex by using the three models illustrated in Fig. 1(a). All simulations were done in the full three dimensional system covering the geometry of the tip in front of the pinhole plate and involve the calculation of multiple individual trajectories of electrons moving in the applied DC field, the time-dependent laser field as well as the Coulomb fields of all the other emitted electrons and all image charges inside the tip (see Methods Section for details). The fields near the tip were computed with including plasmonic effects by solving Maxwell’s equations based on the Multiple Multipole Program^28^.

### Simulations based on re-scattering model

**Figure 2:**
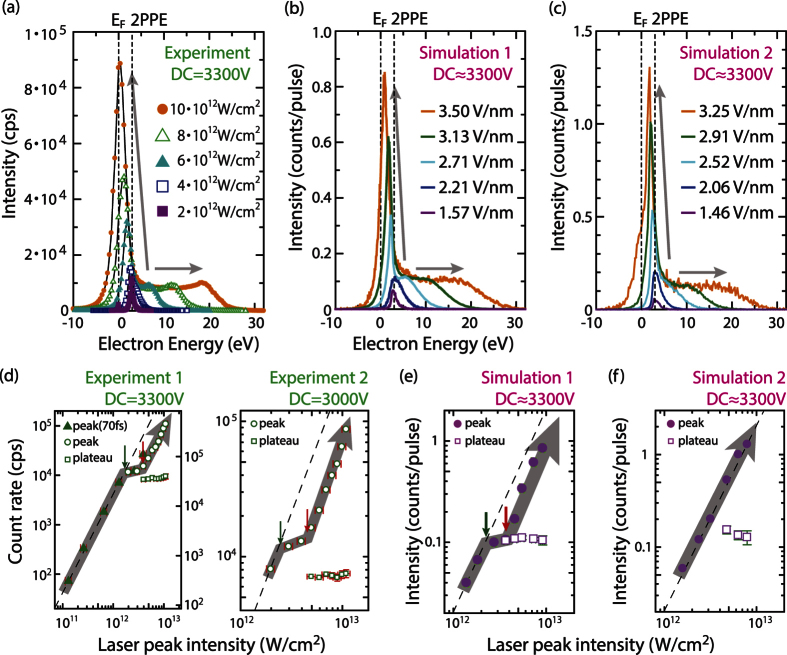
Experimental and simulated energy spectra together with extracted characteristic intensities. (**a**) Experimental and (**b**,**c**) simulated energy spectra for different laser peak intensities. Simulation 1 and 2 are simulated based on the re-scattering model and the transient thermionic emission model, respectively. Corresponding laser fields in the simulations represent the maximum laser fields at the focus without field enhancement. The enhancement factor is 2.4 in our case^3^,^4^. The applied DC voltage has been subtracted from the energy scale such that 0 eV corresponds to the Fermi energy *C*_*F*_ of the tip. (**d**) Evolution of the peak and plateau intensities in the experimental energy spectra for two different DC voltages (open circles), plotted as a function of laser peak intensity including the field enhancement by a factor of 2.4 at the tip apex. Filled triangles in the left panel show peak intensities from an earlier study in the weak-field regime^3^,^4^; their count rates are indicated in the vertical axis at the right side. The scale is adjusted in such a way the level of the data points of the two diffierent experiments become the same around 2 × 10^12^ W/cm^2^. The error bars in peak intensities are due to uncertainty in the beam waist at the focus. (**e**,**f**) Same for the simulated energy spectra. The dashed lines in (**d**–**f**) indicate lines with a slope of 2.

**Figure 3:**
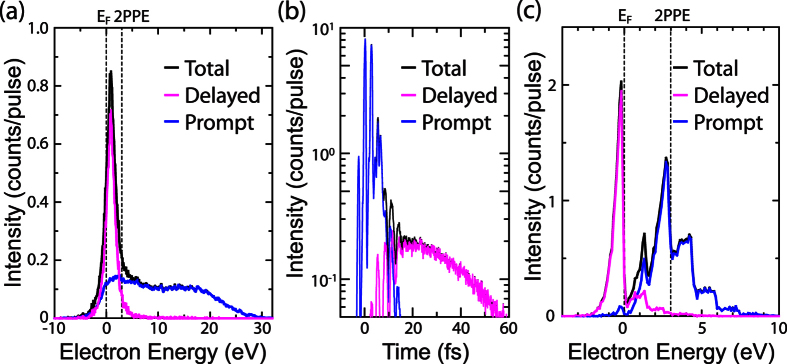
Simulated energy spectra and temporal profiles decomposed into different emission processes. (**a**) Simulated energy spectra at the counter electrode (pinhole plate), (**b**) temporal profiles of the electron emission and (**c**) initial energy spectra on the tip apex before propagation through the vacuum, and thus without distortion by the multi-electron effects. In all panels the spectra are decomposed into prompt and delayed emission processes. The laser field is 3.5 V/nm.

The simulated energy spectra based on Model C are shown in Fig. 2(b). Corresponding laser fields in the simulations are determined by first fitting the simulated spectrum to the experimental one at 6 × 10^12^ W/cm^2^, and then by scaling the laser fields properly with the laser intensities in the experiments. These values were in good agreement with approximate field values that were pre-calculated from the laser intensity and beam waist in the experiments. The resulting spectra reflect the essential details of the experimental observations. The plateau and the peak features, and their evolution with laser intensity, are well reproduced by our simple model. As suggested earlier, the two features originate from prompt and delayed emission, respectively. Figure 3(a) shows the simulated energy spectrum for the maximum laser field at the tip apex, which is 8.4 V/nm including the field enhancement of 2.4, decomposed into contributions from the two emission processes. Clearly, the plateau represents the prompt emission and the peak the delayed emission. The different behaviour can be intuitively understood in terms of the relative strength of the multi-electron effects from a temporal profile of the electron emission as shown in Fig. 3(b): an intense prompt emission within the first 10 fs, closely following the oscillations of the laser field, and a much weaker delayed emission during the next tens of femtoseconds. Both emission currents occur within a 30 × 30 nm^2^ area^3^,^4^. Therefore, the prompt emission is spatially and temporally dense enough to produce the broadened energy distribution due to the strong Coulomb interaction.

Here we would like to point out that the physical origin of the plateau and peak features is different from that of similar features observed in the strong-field regime under conditions where multi-electron effects can be ignored^14^,^15^,^16^,^18^,^19^,^20^,^21^,^29^. There, the plateau is typically modulated with equally spaced peaks (spacing *hν*) due to interferences between electron wave packets resulting from direct emission as well as single and multiple elastic re-scattering off the tip surface, and a signal at low energies represents the direct emission, as discussed by Krüger *et al*.^16^. Similar plateau structures were also observed upon excitation of a tip in the mid-IR, produced by direct acceleration in the subcycle regime^18^. In our regime where the multi-electron effects are dominant, all prompt emission, namely direct emission and elastic re-scattering emission, is broadened into the plateau or its high-energy tail. It should be mentioned that the elastic re-scattering emission is relatively weak in electron signal compared to the direct emission; the previous experiment shows the ratio of the electron signals between elastic re-scattering and direct emission is 0.1 at most^14^,^15^,^16^,^22^. Hence the simulations based on Model C in Fig. 2(b) neglect the elastic re-scattering. The neglect will cause uncertainty in obtained fitting parameters; error bars of laser intensities for Model C in the corresponding figures come from this assumption.

**Figure 4:**
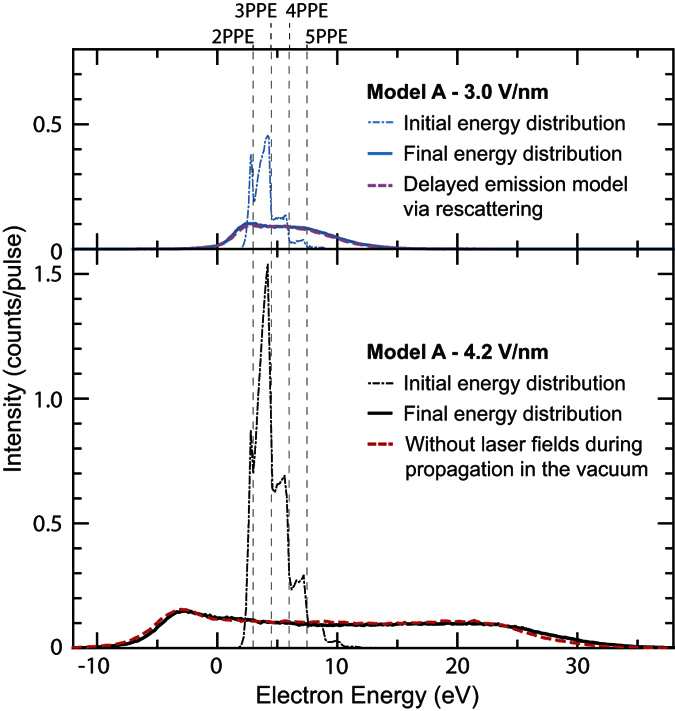
Simulated energy spectra. Energy spectra obtained from Model A of Fig. 1(a) for different laser fields and assumptions. The thin blue and black dashed-dotted curves are the initial energy distribution (see the text). The blue and black solid curves were final energy distributions. The blue and black solid curves were calculated by assuming elastic re-scattering at the tip surface with scattering probability of 1 to keep the number of electrons the same between situations with and without laser fields. The pink dashed curve was calculated by assuming inelastic scattering leading to delayed emission. The red dashed curve was calculated without interaction of the emitted electrons with the oscillating laser field during propagation in the vacuum. The increase in the laser field from 3.0 V/nm to 4.2 V/nm leads to an increase of the emitted charge from 85 e/pulse to 420 e/pulse.

The strong multi-electron effects are illustrated by means of simulations based on Model A. Using this model, rescattering processes are unlikely to occur, as discussed later, and only the plateau feature can be reproduced. Hence it is useful to understand the degree of multi-electron effects in the plateau. The energy spectra for different laser fields are shown in Fig. 4. The number of electrons are in a range similar to the observed values: 85 e/pulse and 420 e/pulse for 3 V/nm and 4.2 V/nm, respectively. In this condition, 3PPE peaks are dominant in the initial energy distributions on the tip surface (the thin blue and black dashed-dotted curves), before electron propagation through the vacuum, *i*.e. before Coulomb interaction distorts the spectrum. The plateau features appear in the final energy distributions at the counter electrode (the blue and black solid curves). With increasing laser field, the spectra broaden in energy while maintaining almost constant plateau height. This is the same behaviour as observed experimentally in the plateau feature. The energy broadening is mainly due to the Coulomb interaction during electron propagation in the vacuum, where the slower electrons get pushed back, the faster electrons pushed forward by the bulk of the emitted electron cloud. The red spectrum of Fig. 4 is simulated without considering the interaction of the emitted electrons with the laser field during electron propagation through the vacuum while keeping the number of the emitted electrons and all the other initial emission conditions the same as in the simulation with the laser field of 4.2 V/nm (the black solid curve). The red and black spectra are almost identical, which means that the formation of the plateau feature is mainly due to the multi-electron effects. The results clearly show the strength of multi-electron effects in the regime where our experiment was done. When multi-electron effects are strong in the prompt emission, whichever emission mechanism in Fig. 1(a) we used, the energy spectra end up with only the plateau feature unless a delayed emission is included. Therefore, we conclude that the delayed emission is necessary to avoid the strong multi-electron effects and reproduce a peak feature.

The simulations based on Model C reveal an intuitive picture of the initial emission mechanisms in the delayed emission process. The initial energy distribution was decomposed into contributions from the prompt and the delayed emission processes as shown in Fig. 3(c). The delayed emission mainly consists of the laser-driven tunnelling emission from the Fermi level, *E*_*F*_. In the delayed emission process, the electrons travelling in the metal after re-entry will typically lose 50 percent of their energy with creating secondary electrons in an inelastic scattering event^30^,^31^. Therefore, the impact energy has to be more than approximately twice the barrier height for the inelastically back-scattered electrons to realize the delayed emission. Because the impact energy depends on the exact time of the initial emission relative to the phase of the driving laser field^32^, only the laser-driven tunnelling emission can produce a significant number of re-colliding electrons with high enough energies owing to its strong dependence on the laser oscillation phase^9^,^12^. In contrast, photo-excited electron emission like in Model A is rather insensitive to the laser phase^9^. This is why Model A cannot reproduce the peak feature, even if we assume inelastic re-scattering for the delayed emission as shown in the upper panel of Fig. 4 (pink dashed curve). Here, only few of the initially emitted electrons are steered back to the surface, and if they are, with low impact energy. Hence, after typically 50 percent of energy loss due to inelastic scattering in the metal, only few can overcome the surface barrier. Model A can thus be excluded as it cannot reproduce the low-energy peak. The delayed emission is therefore attributed mainly to the laser-driven tunnelling emission from the Fermi level, *E*_*F*_. Note that also Model B, considering exclusively laser-driven tunnelling emission, can reproduce the spectra in the strong-field regime quite well, but the 2PPE peak at the lowest laser intensity is not reproduced by Model B. Therefore, only Model C describes the laser intensity dependence of the emission spectra over the whole range of intensities.

The simulations clarify also the physics of the observed kinks in the evolution of the intensity of the low energy peak in Fig. 2(d). Figure 2(e) shows the corresponding plot resulting from the simulated spectra. There are two kinks in the curve, similar to the experimental data. The first kink at the green arrow is due to intensity saturation associated with the multi-electron effects^33^,^34^, as the emission from the 2PPE and higher multi-photon excitation starts to expand towards higher energies and to develop into the plateau feature. After the second kink, marked by the red arrow, the peak intensity increases strongly. This signals the opening of the delayed emission channel due to the laser-driven tunnelling emission, as the impact energies of the electrons on the surface become higher than twice the barrier height. This scenario is further supported by the conventional expression for the maximum impact energy, *I*_*p*_ + 3.2*U*_*p*_, where *I*_*p*_ is the work function and *U*_*p*_ is the ponderomotive energy, a cycle-averaged quiver energy of a free electron in an oscillating laser field^35^. Although the work function is reduced under strong DC fields, here we employed the original work function by following ref. 36. The position of the second kink is at a peak intensity of 4.0 × 10^12^ W/cm^2^ (*U*_*p*_ = 0.26 eV), which corresponds to a maximum impact energy of 5.34 eV, calculated according to this formula. This is approximately twice the effective surface barrier *ϕ*, which is about 2.68 eV for an applied DC voltage of 3300 V producing a DC field of 2.3 V/nm around the emission area. In addition, the transition of the emission mechanism from multi-photoexcitation (model A) to laser-driven tunnelling (model B) is often characterized by the Keldysh parameter, 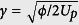. Previous work based on the Schrödinger equation predicted the critical Keldysh parameter to be 2.5 for the transition of the emission mechanism^9^. The strong field regime is below this value. Our simulation shows that the Keldysh parameter is approximately 2.3 at the kink when the delayed emission channel opens, which provides further support for the strong link between the delayed emission channel and the AC-tunneling emission mechanism. Here we would like to point out that, in order to observe the signature of the delayed emission, strong DC fields are helpful because the DC field enhances the emission probability of inelastically re-scattered electrons when they are re-emitted from the surface. On the other hand, the laser fields are no longer important at this stage because the laser pulse has already left the tip region.

**Figure 5:**
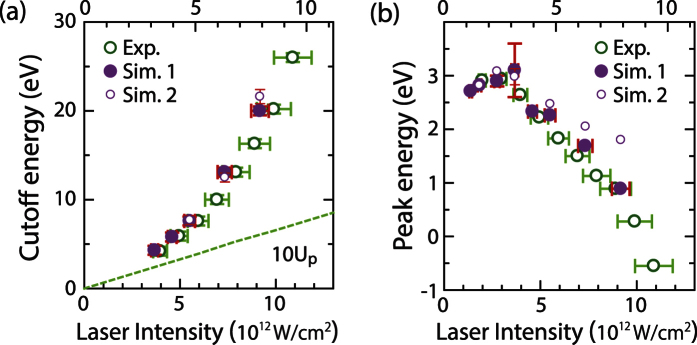
Characteristic energy values extracted from the spectra. (**a**) The cutoff energies of the plateau and (**b**) the energies of the low-energy peak maxima extracted from the experimental and simulated energy spectra are plotted as a function of laser intensity. Simulation 1 and 2 are results from the re-scattering model and the transient thermionic emission model, respectively. The peak intensities for experiments and Simulation 1 are shown in the horizontal axis at the bottom, and the intensities for Simulation 2 are indicated at the upper side. The green dashed line indicates 10*U*_*p*_ expected from the classical cutoff law for ponderomotive energy gain. An example for the cutoff energy of the plateau is indicated by the arrow in Fig. 1(b). Error bars are added whenever the errors are bigger than the symbols.

The evolution of the plateau cutoff energy and the position of the low-energy peak are successfully reproduced by our simulations as shown in Fig. 5(a,b) (Simulation 1). The excellent agreement of the plateau cutoff energies indicates the successful modelling of the multi-electron effects and electron photo-excitation dynamics. It should be emphasized that the cutoff energy increases quadratically with laser intensity, and thereby the cutoff energy largely deviates from the green dashed line of 10*U*_*p*_ expected from the classical cutoff law for ponderomotive energy gain, which underlines once again that the multi-electron effects are significant in our experiment. Experimentally, the laser intensity where the plateau feature starts forming depends on the number of emitted electrons; for lower DC fields the corresponding laser fields have to be higher to establish the plateau feature (not shown). Note that all energy scales for the data shown in Figs 2 and 5 have been corrected for the applied DC voltage bias in order to align the zero energy with the Fermi energy of the sample. Intensity tails at negative energies result from the remaining weaker Coulomb interaction between prompt and delayed electrons, as does the slight shift of the low-energy peak; the slight disagreement between measured and simulated peak shifts seen in Fig. 5(b) is due to the simplified description in our model of the complex physics involved in the delayed emission channel.

### Simulations based on thermionic emission model

We finally address an alternative mechanism that could lead to delayed emission without invoking laser-driven re-scattering. If the photo-excited population of hot electrons would decay more slowly than expected, then this could lead to strong transient thermionic emission^37^,^38^,^39^. In fact, our simulations take thermal effects into account by including electron dynamics inside the tip, such as multi-photon absorption and energy relaxation of the excited electrons due to electron-electron and electron-phonon interactions as well as ballistic transport away from the tip surface (see Methods Section). Our simulations suggest that emission due to hot electrons decays very fast also in the strong-field regime as can be seen in the blue curve of Fig. 3(b). Here, the fast decay is mainly due to the ballistic transport of hot electrons away from the surface^40^, which includes electron emission from the tip. Ballistic transport plays an important role in ultrafast electron dynamics in excited states in metals^40^. Although its effect can be ignored for a very small tip where the size of the apex approximately equals the optical penetration depth^39^, as our tip size is roughly one order magnitude bigger than the penetration depth (20 nm), the ballistic transport should be an important factor. However, the quantitative contribution of the ballistic transport is not clear for our tip. In our ballistic transport model, the electrons moving towards the bulk are assumed to be lost, with no probability to come back to the surface via other scattering processes. This assumption will underestimate the thermal effect^40^. Therefore, we also investigated a model where the transport effect is artificially suppressed by reducing the electron velocities by a factor *C*_*ES*_, thus enhancing the transient thermionic emission.

**Figure 6:**
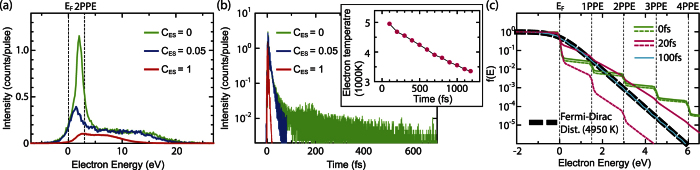
Energy spectra and temporal profiles simulated by the transient thermionic emission model. (**a**) Energy spectra for different electron velocity reduction factors *C*_*ES*_ for ballistic transport and (**b**) corresponding temporal profiles of the electron emission. (**c**) Electron distribution function for various times calculated with *C*_*ES*_ = 0 (solid curves) and *C*_*ES*_ = 1 (dashed curves), where the time zero is defined when the maximum of the carrier envelope of the laser pulse is at the center of the tip apex. After 100 fs, electron distribution function reaches thermal equilibrium and is reasonably fitted by a Fermi-Dirac distribution function as indicated by blue curves. The inset of (**b**) shows evolution of effective temperature extracted from electron distribution functions after 100 fs.

The simulations based on this model cannot reproduce the observed spectra well. Figure 6(a) shows the energy spectra based on Model A for different electron velocity reduction factors *C*_*ES*_ = 1, 0.05 and 0, where all the other parameters are the same as the re-scattering model based on Model C. As expected, when the transport velocities are drastically reduced, a peak feature appears. Without ballistic transport (*C*_*ES*_ = 0), the spectrum appears similar to the experimental one. The resulting temporal profile of the electron emission in Fig. 6(b) shows tails extending to more than 500 fs, which is much longer than for the re-scattering emission shown in Fig. 3(b). The longer tails are due to transient thermionic emission, which can be understood by temporal evolution of electron distribution functions, f(E), in Fig. 6(c). During the first tens of femtoseconds, f(E) resembles nonequilibrium step-like functions in both the thermionic and the re-scattering emission models (solid and dashed curves, respectively). On one hand, excited electron population in the re-scattering model quickly decreases with time due to ballistic transport, which also leads to the quick decay of the prompt emission in Fig. 3(b). On the other hand, excited electron population in the thermionic emission model stays more significant for a longer period and f(E) reaches thermal equilibrium at around 100 fs, where f(E) can be reasonably fitted with a Fermi-Dirac distribution function with an elevated temperature. The evolution of the transient temperatures in the thermionic emission model after 100 fs is plotted as a function of time in the inset of Fig. 6(b). The resulting temperature evolution was similar to the previous work that did not include ballistic transport in their models^37^,^39^. Without ballistic transport, the energy decay channels are established only by electron-electron or electron-phonon couplings in our model. These assumptions are the same as the origins of energy transports in the previous simulations^37^. Therefore, we expect consistency with the previous results. In the thermionic emission model, this slow thermionic emission constitutes the peak feature. Energy spectra with *C*_*ES*_ = 0 are simulated for various laser intensities and shown in Fig. 2(c). Corresponding laser fields in the simulations are determined by the same procedure as Simulation 1 to get the best description of the evolution of the amplitude of the low-energy peak and the plateau cut-off energy in the experiments.

Although at first glance the spectra seem very close to the measured ones (Fig. 2(a)), they do not reproduce three observed characteristics. First, the peak shape is different. Specifically, the connecting region where peak and plateau meet (around 5 eV) is smeared out, especially for lower laser intensities. At 2.52 V/nm in Fig. 2(c), the plateau structure is more like a shoulder of the peak, while it is a clear plateau in the corresponding measured spectrum (spectrum for 6 × 10^12^ W/cm^2^ in Fig. 2(a)). Second and more importantly, the evolution of the peak intensities with varying laser intensities in Fig. 2(f) shows no kinks like those seen in Fig. 2(d,e), and thus does not agree with the experimental results. In the transient thermionic emission model, the delayed emission exists even for the weak field regime. Hence, we cannot expect the sudden opening of the new delayed emission channel like in the case of the laser-driven tunnelling process. Note that the two kinks could not be reproduced by this model even when varying the DC field. Third, although the plateau cutoff energy is well reproduced by the thermionic emission model as shown in Fig. 5(a), the energies of low-energy peak deviate substantially from the observed values (Fig. 5(b)) (Simulation 2). This disagreement is due to very slow delayed emission beyond 50 fs, which leads to weaker Coulomb interaction between the prompt and the delayed electrons. In Fig. 5, the horizontal axis of the upper side indicates the peak intensities of Simulations 2. Their scale are adjusted in such a way that the corresponding data points of two different simulations can be compared. The determined laser peak intensities of Simulation 2 are approximately 20 percent lower than the corresponding experimental values; their deviations are beyond the experimental errors. In addition, since we need to completely neglect ballistic transport (*C*_*ES*_ = 0) to have the low-energy peak, we conclude that transient thermionic emission is not a main contributor to the observed phenomena. According to previous experimental work^39^, thermionic emission can be observed above a peak intensity of 1 × 10^13^ W/cm^2^ under strong THz fields ranging from 1 GV/m to 10 GV/m while the delayed emission channel is opened around 4.0 × 10^12^ W/cm^2^ under DC fields of approximately 2.5 GV/m in our case. Since the frequency of the THz radiation is one order of magnitude lower than that of the near-infrared (NIR) laser pulses^39^, THz fields can be considered as a quasi-static field during the irradiation with NIR pulses. If the effect of THz fields is the same as that of DC fields, thermionic emission cannot be expected for our experimental condition.

Finally, we would like to comment on the thermionic emission contribution to the low-energy peak. As we mentioned above, the ballistic transport contribution would be overestimated at *C*_*ES*_ = 1 because of our simple model. The actual ballistic transport effect would be effectively determined by *C*_*ES*_ which is in the range between 1 and 0. As seen above, the smaller the *C*_*ES*_ is, the more thermionic contributions can be found at low energies. Hence, if the actual *C*_*ES*_ is less than one, the low-energy peak should contain more thermionic components in the re-scattering model. In the observed spectrum at the laser peak intensity of 1 × 10^13^ W/cm^2^ in Fig. 2(a), we find that the low-energy peak constitutes 48% of the integrated emission spectrum. Based on the above argument, the low-energy peak would contain a certain amount of thermionic emission. This, however, will not affect our conclusion because thermionic emission alone could not reproduce the observed signatures. Especially the two kinks we observed in Fig. 2(d) cannot be explained so far unless we introduce the re-scattering model.

## Summary

In summary, our observations and simulations strongly suggest the opening of a delayed emission channel in strong fields. The suggested delayed emission is associated with electrons emitted via laser-driven tunnelling emission, mainly from the Fermi level. These emitted electrons are driven back into the metal by the oscillating laser fields, and some of the electrons reappear in the vacuum with some delay time after undergoing inelastic scattering and cascading processes inside the metal. The delayed emission manifests itself by a prominent low-energy peak. The rapid growth of this peak with laser intensity reflects the transition from the multi-photon to the tunneling emission regime. The delayed emission provides information on the attosecond dynamics of electron tunnelling at a solid surface, as well as on the femtosecond scale electron scattering processes within the tip material. Moreover, the understanding of these processes as demonstrated here should be useful in designing electron sources with attosecond temporal confinement. One of the more daring ideas is to use a carbon nanotube with a closed end on one side and an open end on the other side. Illuminating the closed end with strong-field laser pulses should lead to the electron emission from the open end on the other side. The emission from the open end should be still in phase with the oscillation of the laser fields driving the electron emission because the electrons re-entering the nanotube would be transported ballistically. Thus we should be able to extract the majority of the laser-driven tunnelling emission at the opposite end of the nanotube, most of which is absorbed within the metal in case of a tungsten tip via inelastic scattering processes^22^. Such a pulsed electron source should find applications in ultrafast science, particularly impacting cutting-edge technology that relies on bright and coherent electron beams like electron diffraction, microscopy, or holography^41^,^42^,^43^,^44^.

## Methods

### Experimental

A tungsten tip is mounted inside a ultra-high vacuum chamber (9 · 10^−11^ mbar). The apex of the tungsten tip is crystallized and oriented towards the [011] direction; its radius of curvature is approximately 100 nm. Laser pulses are generated by an oscillator (centre wavelength: 830 nm; repetition rate: 80 MHz; pulse duration: 7 fs) and introduced into the vacuum chamber. By means of spectral phase interferometry for direct electric-field reconstruction (SPIDER) outside of the vacuum chamber, we confirmed that the pulse width can reach 7 fs. A parabolic mirror in the chamber focuses the laser to approximately 3.5 *μ*m diameter (1/*e*^2^ radius) onto the tip apex. The tip was mounted on a 5-axis piezo stage controlling three Cartesian coordinate positions x, y, z, as well as a tilt angle 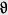 and an azimuthal angle *φ* around the tip axis. The tip apex can be precisely positioned into the focus of the laser by using the piezo stages. Linearly polarized laser light was used, with the polarization vector parallel to the tip axis. A pair of chirped mirrors is used for dispersion compensation. To achieve the shortest duration of the laser pulses at the tip apex, the positions of the glass wedges were optimized in such a way that the electron intensity from the tip was maximized.

**Figure 7:**
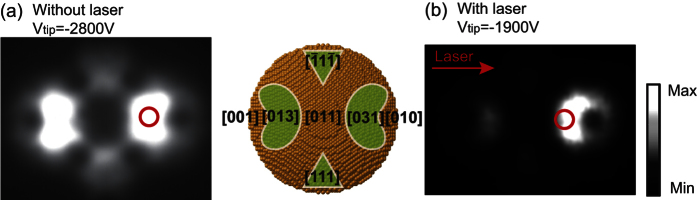
Electron emission patterns from the tungsten tip. (**a**) Electron emission patterns without laser illumination, measured with a tip voltage *V*_*tip*_ of −2800 V. The inset of (**a**) shows the front view of the atomic structure of the tip apex based on a ball model, in which green areas with white edges indicate the emission sites. (**b**) A laser-induced field emission pattern. The tip voltage *V*_*tip*_ was −1900 V and the laser peak intensity was 1 × 10^12^ W/cm^2^. Red circles indicate positions of the pinhole during measurements of the energy spectra.

To evaluate intensity and energy distributions of the electron emission, there are two types of detectors installed in the vacuum chamber; one is a two-dimensional detector and the other one is a hemispherical energy analyzer (Vacuum Generators CLAM2). The two-dimensional detector was used to observe the electron emission patterns from the tip. Cleanliness of the tip apex can be assessed from the emission patterns^45^. If the surface is clean, we can observe the emission pattern shown in Fig. 7(a). The intensity distribution of the electron emission is mainly dependent on the distribution of the local work function on the apex^3^. Hence, the most intense emission can be observed around a [310] type facet as schematically drawn in the inset. A clean tip surface was prepared by flash-annealing the tip. Since the tip apex can be quickly contaminated even under ultra-high vacuum conditions, all the measurements were done within 15 minutes after the sample heating.

The electron analyzer is used to measure energy spectra of the electrons emitted from the tip apex. A pinhole plate covered with phosphor was mounted between the tip and the analyzer in order to observe emission patterns and to define a particular emission site to be measured. The diameter of the pinhole is 2 mm and the distance between the pinhole plate and the tip apex is 13 mm. In these experiments, the pinhole is positioned on the [310] type facet; its position is roughly indicated by a red circle in Fig. 7(a). Note that the laser-induced electron emission shows an asymmetric emission pattern as shown in Fig. 7(b). This is due to plasmonic effects, which was already explained in our previous work^3^,^4^. The pinhole is positioned at the most intense emission site. Excellent performance of the analyzer system in terms of energy resolution was already demonstrated in our previous work^6^,^7^. The measuring time for each energy spectrum is about 2 min. During this time, the tip position is stable. The whole vacuum chamber is mechanically stabilized by air dampers and made of *μ*-metal for magnetic shielding.

### Simulation of energy spectra

The energy spectra of the laser-induced electron emission were reproduced by simulating optical fields at the tip, optical excitation, emission from the tip surface and propagation through the vacuum with taking the space charge effects into account. All the simulations were done in the full three dimensional system.

### First step: calculation of optical fields

In the first step, we simulated the time evolution of the optical local fields on the tip apex when the 7 fs laser pulse is passing by the apex of the tungsten tip. For this simulation, we used the software package OpenMaXwell for solving Maxwell’s equations based on the Multiple Multipole Program^28^. The optical fields are simulated in the frequency domain. 32 equally spaced frequencies were selected within a band covering an experimentally obtained laser spectrum, and the optical fields are simulated for each frequency. A laser pulse was constructed by superimposing the resulting optical fields of the 32 different frequencies weighted by the square root of the intensity of the measured laser spectrum at a given frequency.

### Second step: calculation of emission current distribution

In the second step, the simulated local fields on the tip apex were used to calculate the emission current distribution on the apex as was done previously by using the Fowler-Nordheim theory^3^,^4^,^6^,^7^. The current density *j*_*calc*_ of the tunnelling emission can be described based on the free-electron model as follows^44^,^46^,^47^,^48^,


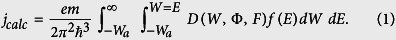


Here, *e* is the electron charge and *m* is an effective electron mass, *−W*_*a*_ is an effective constant potential energy inside the metal, W is the normal energy with respect to the surface, and E is the total energy. Important factors are *D*(*W*, Φ, *F*) and *f*(*E*). *D*(*W*, Φ, *F*) is the probability that an electron with the normal energy W tunnels through the surface barrier. It depends exponentially on the potential barrier above W, the area of which is determined by the work function Φ and the electric field *F*. The work function on the tip apex was extracted from the experimentally obtained emission intensity distributions from the tip. We already obtained such a work function map in our previous work^3^,^4^ and used the same values here. *f*(*E*) is an electron distribution function.

By using the Eq. (1), we can calculate the emission current for all models. In the case of multi-photon excitation as shown in model A of Fig. 1(a), transient electron distribution functions, which are explained below, have to be substituted for *f*(*E*) in Eq. (1) and *F* = *F*_*DC*_, where *F*_*DC*_ is the applied DC field. In the case of laser-driven tunnelling emission defined as model B, we have *F* = *F*_*DC*_ + *F*_*AC*_, where *F*_*AC*_ is the enhanced optical field on the tip apex, and *f*(*E*) is the Fermi-Dirac distribution. In the case of model C, which is a combination of models A and B, the transient electron distribution functions are used for *f*(*E*), and *F* = *F*_*DC*_ + *F*_*AC*_.

To simulate transient electron distribution functions in metals irradiated with a laser pulse, we mainly follow Rethfeld’s approach^49^,^50^. Electron-electron (*e-e*) interaction, electron-phonon (*e-p*) interaction, energy absorption from the laser pulse and additionally energy transport due to ballistic transport^40^,^51^,^52^,^53^ are included in a system of Boltzmann’s equations to obtain the temporal evolution of the distribution function of the electron gas *f*(***k***, ***z***) and the phonon gas *g*(***q***),


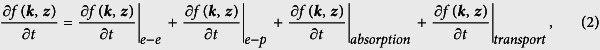



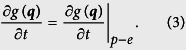


Here, ***k*** and ***q*** are momenta of electrons (e) and phonons (p), respectively. To include the transport effects, we use a spatially dependent electron distribution function, where z is the direction perpendicular to the surface. The laser is assumed to penetrate into the metal with a penetration depth of 20 nm, and thereby the excited electron distribution has a spatial dependence in the z direction. The rate of energy absorption is calculated at the topmost surface. The evolution of *f*(***k***, ***z***) due to *e* − *e* and *e* − *p* scattering are also calculated at the topmost surface and *f*(***k***, ***z***) was normalized at the surface values. We assumed ballistic electron transport as main channel for the energy transport because of the ultrashort time scale^40^,^51^,^52^,^53^, and used the velocities of electrons propagating perpendicular to the surface in the metal, *v*_⊥_^51^. After each time step Δ*t*, *f*(***k***, ***z***) was moved towards the surface with a distance of *v*_⊥_Δ*t*. When the electrons are at the topmost surface, they will either be emitted from the surface or be reflected elastically by the surface potential barrier.

It should be mentioned that these interaction terms in Eq. (2) are written without any relaxation-time approximation. To calculate *e-e* and *e-p* scattering, we used free-electron and Debye models with a Fermi energy of 9.2 eV^54^, an effective electron mass of 7.7 · 10^−31^ kg, a Debye temperature of 400 K^55^, and a speed of sound of longitudinal phonons of 5220 m/s^56^. The effective mass was determined in such a way that the density of valence electrons per unit cell corresponds to that of tungsten; tungsten has two valence electrons in its unit cell. A time step of 0.1 fs was used for simulating the time evolution of the electron distribution functions. The 7 fs laser pulse is moved in 0.1 fs steps typically from −10 fs to 30 fs across the tip apex. Time zero is defined by the instant when the maximum of the envelope of the laser pulse meets the top of the apex. A grid of approximately 2300 points are generated on the tip surface, and transient electron distribution functions are calculated on each point.

### Third step: calculation of electron trajectories

In the third step, the electron trajectory simulation was performed. The total yield is obtained by integrating the calculated emission current over energy, time and space. The number of electrons per pulse was 850 at the highest laser intensity. The initial electron emission times, energies, positions and directions were determined based on the Monte Carlo method by using the calculated emission current distribution. The tunnelling emission was delayed by the Keldysh time, *τ*_*K*_, multiplied by a factor *C*_*τ*_. Here, *τ*_*K*_ is expressed as *τ*_*K*_ = *γT*_0_/4*π* where *γ* is the Keldysh parameter and *T*_0_ is the laser oscillation period^57^. The Keldysh time varies with position and time of electron emission because it depends on the instantaneous laser fields and the effective barrier height reduced by DC fields. With this model, we effectively determined the start position and time after tunnelling dynamics. Whether the emitted electrons can be driven back to the surface by the oscillating laser field depends on the tunnelling delay time in our model. *C*_*τ*_ was adjusted in such a way that the largest number of electrons is redirected to the surface with the highest energy. This condition resulted in a value of 1.4 for *C*_*τ*_ for the case of Model C. If *C*_*τ*_ is set to zero then few electrons are re-directed towards the surface. In addition, the start velocities of the tunnelling electrons are calculated from the electron velocities inside the metal; the perpendicular component of the velocity with respect to the surface is set to zero when the electrons are at the tunnelling exit, and additionally we added a velocity change due to the vector potential experienced during the delay time. It should be noted that the time-dependent Schrödinger equation should be employed for accurate theoretical description of optical tunneling time as discussed in refs 9 and 10.

After determining the initial conditions, the electrons were propagated through the vacuum, where they experience four kinds of forces: 1. laser fields, 2. DC fields, 3. Coulomb forces between electrons (space charge effects), and 4. image charge forces from the tip surface. For calculating DC fields, we used the OpenMaXwell static solver with fictitious charges along the tip axis^28^, which is basically the same technique as explained in ref. 58. The tip is biased at approximately −3300 V and there is a grounded counter electrode 13 mm away from the tip apex, which represents the conditions of the experimental setup. The DC fields are set to 2.5 V/nm at the very top of the tip apex, which is a reasonable value when tunnelling emission due to the DC fields is observed. For simulating dynamics of image charges in the tip, we simulated the positions and amounts of classical image charges for a metallic sphere. The simplified spherical approximation of the tip is acceptable because image forces are important only very close to the tip apex. Tracking electron trajectories was done with including those image charges in the computational fields, and calculated Coulomb forces between all the electrons and image charges.

During propagation, the time steps were dynamically determined in such a way that the mean energy change of all the electrons for each time step was a constant. This constant determines the accuracy of the simulation; the cumulative absolute energy error is estimated to be about ±0.2 eV. The tracking-electron simulation stops when all the emitted electrons reach the counter electrode (pinhole plate). This entire process was repeated until enough statistics was obtained.

The electrons which are re-scattered from the tip experience the delay processes as illustrated in Fig. 1(b). In electron travelling in the metal, we assume a simple model where the electrons travel a distance of the order of twice the inelastic mean free path before reappearing in the vacuum. Here we applied an effective inelastic scattering rate when electrons have re-entered the surface. This rate is a global fitting parameter in our simulation, i.e. one common value for all laser intensities, which is adjusted in order to best describe the peak-to-plateau intensity ratios from the experimental data. The physics behind this quantity is complicated and includes contributions from inelastic forward and backscattering in the solid and also creation of secondary electrons^31^,^59^,^60^. We obtained a value of 0.45 for best fit, and the number of electrons reappearing in the vacuum is effectively determined simply by using this inelastic scattering rate. Note that the elastic scattering probability is around 0.1–0.15 surface in our electron energy regime^60^. Due to the concomitant creation of secondary electrons via cascading processes, the effective inelastic scattering rate at low energies can be higher than the elastic scattering rate, and a value of 0.45 appears still physically sound. Regarding elastic scattering, the typical time scale for travelling in the metal is very short, of the order of 1 fs or less for tungsten in our energy regime^27^. Hence elastically re-scattered electrons can be considered as prompt, they contribute to the plateau feature or its high-energy tail. As discussed in the text, we neglected these processes.

The delay times due to the inelastic re-scattering processes were calculated by dividing the electron travelling length by the group velocity, taken from a free-electron model and using the effective mass of tungsten, for the round trip from and back to the surface. To determine the path length which electrons travel before the inelastic scattering takes place, we assume that the scattering events are stochastic. The probability distribution follows the Poisson distribution with its maximum positioned at the inelastic mean free path (IMFP) of tungsten^61^. The IMFP is obtained from ref. 27. The thus calculated delay time is multiplied with a factor *C*_*l*_ to have an evolution of the peak feature similar to the observations. The resulting *C*_*l*_ was 1.8. This high value is considered to be due to the particularly low group velocity near energies of 5 eV above the Fermi level as calculated from dispersion relations^27^.

### Fourth step: calculation of energy spectra

In the fourth step, the energy spectra are calculated from the final velocities of the electrons which enter the pinhole of the electrode, as illustrated in our experimental setup in Fig. 1(b). Note that we applied a five-point moving average to smooth the obtained spectra representing the prompt emission, *i*.e. the plateau. No smoothing was done for the delayed emission, *i*.e. the low-energy peak.

### Summary of free parameters

In our simulations in the second and the third steps, we used four free parameters. They are summarized below.

1. Multiplication factor for tunneling time, *C*_*τ*_: The value is 1.4.
2. Multiplication factor for re-scattering delay time, *C*_*l*_: The value is 1.8.
3. Effective inelastic scattering rate in the metal: The value is 0.45.
4. Multiplication factor for electron velocities in ballistic transport, *C*_*ES*_: If it is 0, ballistic transport is completely ignored and we call it thermionic emission model. If it is 1, ballistic transport significantly cools the excited electron gas and we used this condition for the re-scattering model.

## Additional Information

**How to cite this article**: Yanagisawa, H. *et al*. Delayed electron emission in strong-field driven tunnelling from a metallic nanotip in the multi-electron regime. *Sci. Rep.* **6**, 35877; doi: 10.1038/srep35877 (2016).

**Publisher’s note:** Springer Nature remains neutral with regard to jurisdictional claims in published maps and institutional affiliations.
